# Redox imbalance drives magnetic property and function changes in mice

**DOI:** 10.1016/j.redox.2025.103561

**Published:** 2025-02-21

**Authors:** Chuanlin Feng, Lei Zhang, Xiaoyuan Zhou, Shiyu Lu, Ruowen Guo, Chao Song, Xin Zhang

**Affiliations:** a High Magnetic Field Laboratory, CAS Key Laboratory of High Magnetic Field and Ion Beam Physical Biology, Hefei Institutes of Physical Science, Chinese Academy of Sciences, Hefei, 230031, China; b Science Island Branch of Graduate School, University of Science and Technology of China, Hefei, 230026, China; c Institutes of Physical Science and Information Technology, Anhui University, Hefei, 230039, China

**Keywords:** Magnetic properties, Magnetic susceptibility, ROS, Iron metabolism, Magnetic fields

## Abstract

The magnetic properties of substances directly determine their response to an externally applied magnetic field, which are closely associated with magnetoreception, magnetic resonance imaging (MRI), and magnetic bioeffects. However, people's understanding of the magnetic properties of living organisms remains limited. In this study, we utilized *NRF2* (nuclear factor erythroid 2-related factor 2) deficient mice to investigate the contribution of redox (oxidation–reduction) homeostasis, in which the key process is the transfer of electron, a direct target of magnetic field and origin of paramagnetism. Our results show that the *NRF2*^*−/−*^ mice exhibit significantly altered systemic redox state, accompanied by increased magnetic susceptibility, particularly in the liver and spleen. Further analyses reveal that the levels of paramagnetic reactive oxygen species (ROS) in these tissues are markedly elevated compared to wild-type mice. Moreover, the concentrations of Fe^2+^ and Fe^3+^ are significantly elevated in *NRF2*^*−/−*^ mice, which are directly correlated with the increased magnetic susceptibility. The disrupted redox balance in *NRF2*^*−/−*^ mice not only exacerbates oxidative stress and iron deposition, but also induces impairment to the liver and spleen. The findings highlight the combined effects of ROS and iron metabolism in driving magnetic susceptibility changes, providing valuable theoretical insights for further research into magnetic bioeffects and organ-specific sensitivity to magnetic fields.

## 1 Introduction

Life on Earth has evolved in a magnetic field environment, and many organisms possess the ability to sense magnetic fields [1]. This ability may arise from the influence of magnetic fields on the quantum spin states of paramagnetic free radicals, thereby eliciting biological effects [[2], [3], [4]]. On the other hand, free radicals, as intermediates in redox reactions, play pivotal roles in multiple physiological and pathological processes because redox reactions drive energy conversion in biological systems through electron transfer, forming the foundation of essential processes such as cellular metabolism and photosynthesis [5]. It has long been known that excessive accumulation of free radicals can lead to oxidative stress, cellular damage and disease [6,7]. And magnetic fields can modulate redox states and antioxidant capacity in organisms [[8], [9], [10], [11], [12]]. However, how redox states affect the magnetic properties of organisms is still unclear.

Magnetic susceptibility, a key magnetic property parameter describing a material's ability to be magnetized in an external magnetic field, is critical for understanding biological responses to magnetic fields. In other words, it quantifies the material's response to an applied magnetic field, indicating how easily it can be magnetized and how much it distorts the magnetic field. Previous studies have shown that static magnetic fields (SMFs) are generally safe for healthy mice [13,14], but can generate variable effects in mice with liver-related metabolic diseases [8,12,15,16]. For example, a 0.1–0.2 T near homogenous SMFs continuous exposure to 1.7 years doesn't generate any noticeable harm to healthy mice [17], but exerts significant effects on liver with diseases when exposed for only a couple of days to a few weeks [16]. Moreover, the spleen and liver of mice with severe diabetes also exhibit significantly higher sensitivity to high-field gradient SMFs exposure than other organs [18]. The tissue-specific magnetic property changes will provide valuable information for the seemingly inconsistent data about magnetic field bioeffects in the literature.

Magnetic property changes also provide important references for magnetic susceptibility-based MRI diagnosis techniques including quantitative susceptibility mapping (QSM). Materials with different magnetic susceptibilities cause local magnetic field inhomogeneities. Although magnetic signals from biological samples are typically weak, differences in magnetic susceptibility provide valuable insights for medical diagnosis and tissue sensitivity to magnetic fields. The relative magnetic susceptibility of different tissues can be estimated and calculated based on MRI data in susceptibility-weighted imaging (SWI) [19] and QSM [20,21], which can provide non-invasive spatially resolved information about the relative magnetic susceptibilities of different tissues in vivo. However, they are calculated based on the phase shifts in MRI data, which involve complex post-processing and calibration, and their accuracy depends on the quality of the MRI data, the reconstruction process, as well as reference values for specific tissue magnetic susceptibility to interpret results [[22], [23], [24]]. In contrast, superconducting quantum interference device (SQUID) can directly provide very accurate and direct measurement of the absolute magnetic susceptibility values of tissue, cells, and even molecules of very small sample size. It offers higher sensitivity, requires no complex post-processing, allows for direct absolute quantification of small samples, and makes it an ideal tool for accurate magnetic susceptibility measurements for mechanism studies [25]. Apparently, in medical and clinical fields, MRI-based techniques are often more suitable, but when detailed, precise and absolute magnetic susceptibility measurements of small samples are needed, SQUID will be more ideal [[26], [27], [28]]. Notably, the absolute magnetic susceptibility values obtained by SQUID provide critical information for the interpretation of QSM and SWI results.

However, the exact contribution of factors other than iron, such as free radicals, to the magnetic susceptibility of biological samples under physiological and pathological conditions still remains unclear, hindering a more comprehensive understanding of the relationships between magnetic properties, redox states, free radical levels, and biological functions. The challenge is further compounded by the short lifespan and low concentration of free radicals, as well as the influence of factors such as water diamagnetism, biomolecular paramagnetism, and local ferromagnetism caused by iron overload, all of which affect the magnetic properties of biological samples. These intertwined factors amplify the complexity of studying the link between magnetic susceptibility and redox physiological function.

To elucidate the relationships between magnetic properties, redox states, free radical levels, and biological functions, we employed *NRF2* knockout mice. *NRF2* plays a critical role in maintaining redox balance [[29], [30], [31]] and is integral to regulating systemic iron homeostasis [[32], [33], [34]]. Our results demonstrate that *NRF2* deficiency not only induces significant alterations in the systemic redox state of mice, but also increases ROS and iron content in the liver and spleen, which synergistically lead to increased magnetic susceptibility and tissue damage in these organs.

## 2 Materials and methods

### 2.1 Animal models and sample collection

All animal welfare and experimental procedures were performed strictly according to the *International Guiding Principles for Biomedical Research Involving Animals*. All animal procedures were approved by the Institutional Animal Care and Use Committee of Hefei Institutes of Physical Science, Chinese Academy of Sciences (SWYX-DW-2021-22). Male wild-type (C57BL/6N) mice and *NRF2*^−/−^ mice were purchased from GemPharmatech Co., Ltd. (D000274, Nanjing, China) and Cyagen Co., Ltd. (S–KO-03360, Suzhou, China), respectively. After a one-week acclimation period, all mice were maintained in a specific pathogen-free (SPF) environment with a temperature of 22°C–24 °C, 60 % ± 10 % relative humidity, and a 12-h light/dark cycle. Prior to individual experiments, each animal was assessed for suitability as required by animal welfare regulations.

All experiments were performed on *NRF2*^*−/−*^ and WT mice with identical procedures side-by-side. After completing blood glucose, glycated hemoglobin (HbA1c), glucose tolerance, and insulin tolerance tests, all mice were euthanized to collect blood and tissues. The blood was centrifuged at 3500×*g* for 15 min to separate serum. The tissues were harvested using ceramic tools, weighed, and immediately subjected to magnetic susceptibility measurements, followed by frozen or fixed in 4 % paraformaldehyde for other analysis.

### 2.2 SQUID measurement

The Quantum Design magnetic property measurement system 3 (MPMS3) SQUID magnetometer (MPMS3, Quantum Design, USA) was used to evaluate the magnetic properties of the samples. Before the experiment, weighed fresh mice organs, 150 μL FeCl_2_ solution (450936, Sigma, USA) (prepared in a nitrogen atmosphere glove box), 150 μL FeCl_3_ solution (701122, Sigma, USA) (prepared in a nitrogen atmosphere glove box), 150 μL ferritin solution (F4503, Sigma, USA), 150 μL NaCl solution (S9888, Sigma, USA), and 150 μL H_2_O (ultrapure water) were loaded into the sample holder (8505-013 (C130D), Quantum Design, USA) and vacuum-sealed. M − H (magnetization―magnetic field) curves were measured at 300 K, with a magnetic field scanning range of −10000 Oe to 10000 Oe, and measurements taken at 2500 Oe intervals. The sample holder was used to correct for sample magnetic susceptibility. The magnetic moment of the sample was obtained by subtracting the magnetic moment signal of the holder. The mass magnetic susceptibility (χ_(mass)_) of the material in an external magnetic field was calculated using the equation:

$$\chi_{\left(\text{mass}\right)}=M/\rho H=\mu /\text{mH}$$

where M is the magnetization, ρ is the mass density of sample, μ is the magnetic moment, H is the magnetic field strength, and m is the mass of sample. At each measurement point, the magnetic moment was divided by the sample mass to calculate the mass magnetization (M) per unit mass. All M and H data were fitted linearly in MATLAB R2022a using SquidLab software to generate the M − H curve, where the slope represents the sample's magnetic susceptibility value [35]. For the M − H curve, four data points were collected at each non-zero magnetic field and six at zero field for each sample. The M − H curve was obtained through linear fitting.

### 2.3 Blood glucose and HbA1c measurement

Blood glucose levels of the mice after fasting were measured via tail-vein blood sampling using a blood glucose meter (ACCU-CHEK Active@ 378, Roche, Ireland). HbA1c levels were measured using a glycated hemoglobin analyzer (A1CNow+, Sinocare, China).

### 2.4 Real-time quantitative PCR

Total RNA was extracted from tissue using Trizol reagent (15596018, Thermo Fisher Scientific, USA). The RNA was then reverse transcribed into cDNA using a cDNA synthesis kit (R312, Vazyme, China) following the manufacturer's instructions. The *NRF2* primer sequences are: forward: AGCACAGCCAGCACATTCTCC; reverse: GACCAGGACTCACGGGAACTTC, the *NQO1* primer sequences are: forward: AGCTGGAAGCTGCAGACCTG; reverse: CCTTTCAGAATGGCTGGCA, and the *β-actin* primer sequences are: forward: GGCTGTATTCCCCTCCATCG; reverse: CCAGTTGGTAACAATGCCATGT. Real-time quantitative PCR was performed on a Light Cycler 96 (Roche Diagnostics GmbH, Switzerland) using SYBR Green Mix (Q311, Vazyme, China). Relative quantification was achieved using the ΔΔCt method, normalized to *β-actin*.

### 2.5 Glucose and insulin tolerance test

Glucose tolerance test (GTT) and insulin tolerance test (ITT) were performed on the mice. Mice were intraperitoneally injected with 1 g/kg glucose (G7528, Sigma, USA) or 0.75 U/kg insulin (Novo Nordisk, Denmark). After injection, blood glucose levels were measured via tail vein sampling using a blood glucose meter (ACCU-CHEK Active@ 378, Roche, Ireland) to assess changes in glucose levels. The area under the curve (AUC) was calculated to quantify GTT and ITT levels.

### 2.6 Histological analysis (hematoxylin and eosin, picrosirius red, and oil red O)

Mice tissue was fixed in 4 % paraformaldehyde and subsequently embedded in paraffin. Paraffin-embedded samples were sectioned and stained with hematoxylin and eosin (H&E), oil red O, and picrosirius red. Images were acquired using a microscope, and quantitative analysis was performed using Fiji software (National Institutes of Health, USA).

### 2.7 DAB-enhanced Prussian blue staining

Prussian blue reacts with iron in the tissue to form ferric ferrocyanide, clearly marking the distribution of iron. Additionally, by using DAB (3,3′-diaminobenzidine) as a chromogenic substrate, the contrast and intensity of the staining signal are further enhanced, improving the sensitivity of iron detection. Specifically, the tissue fixed in 4 % paraformaldehyde was washed with PBS, sectioned, and incubated in freshly prepared Perls' solution (5 % potassium ferrocyanide/10 % hydrochloric acid) for 1 h, followed by a 15 min incubation in DAB. Quantification was performed using Fiji software (National Institutes of Health, USA).

### 2.8 Immunohistochemical analysis (F4/80, CD86, and 4-hydroxynonenal (4-HNE))

The liver and spleen were fixed in 4 % paraformaldehyde, dehydrated in a graded ethanol series and xylene, and then were embedded in paraffin. Paraffin-embedded samples were sectioned for immunohistochemistry analysis. Tissue sections were treated with 3 % H_2_O_2_ followed by antigen retrieval using 10 mmol/L sodium citrate buffer (pH 6.5). Non-specific antibody binding was blocked by 1 % bovine serum albumin occlusion at room temperature for 1 h. Sections were incubated overnight at 4°C with primary antibodies: anti-CD68 (28058-1-AP, Proteintech, China), anti-F4/80 (28463-1-AP, Proteintech, China), or anti-4-HNE (bs-6313R, Bioss, China). Following three washes with TBST, the sections were incubated with HRP-conjugated goat anti-rabbit IgG secondary antibody (ab6721, abcam, USA) for 1 h at room temperature. After DAB staining, the sections were counterstained with hematoxylin, dehydrated, mounted, and photographed using a microscope. 4-HNE is generated through the peroxidation of polyunsaturated fatty acids under the action of free radicals, and it can react with proteins, DNA, and other cellular macromolecules to form adducts, especially with the thiol groups (SH) of proteins, leading to Michael addition modification products that influence protein function and cause cellular dysfunction [[36], [37], [38]]. Based on previous studies [[39], [40], [41]], the adducts formed by 4-HNE can be detected and quantified through immunohistochemistry. Therefore, we detected signals corresponding to proteins modified by this compound. Quantification of the positive areas was performed using Fiji software (National Institutes of Health, USA).

### 2.9 TdT-mediated dUTP Nick-End labeling (TUNEL) staining

The TUNEL assay was performed according to the manufacturer's instructions using a TUNEL kit (C1098, Beyotime, China). Briefly, the tissue was fixed with 4 % paraformaldehyde and then embedded in paraffin. Paraffin-embedded tissue sections were deparaffinized and hydrated, followed by permeabilization with proteinase K. After incubation with 3 % H_2_O_2_ at room temperature for 10 min, the slides were covered with terminal deoxynucleotidyl transferase (TdT) reaction solution and incubated in a humidified chamber at 37 °C for 60 min. Next, the sections were incubated with the working solution of streptavidin-HRP for 30 min at room temperature. The slides were stained with DAB for 15 min at room temperature, followed by hematoxylin counterstaining. After microscopic imaging, quantification was performed using Fiji software (National Institutes of Health, USA).

### 2.10 Dihydroethidium (DHE) staining

The frozen tissue was sectioned (4–5 μm), immersed in 1 % acetone fixation solution at room temperature for 5 min, followed by PBS washing. The sections were incubated with 10 μM DHE (309800, Sigma, USA) for 1 h, then washed with PBS and imaged using a confocal microscope (SpinSR10, Olympus, Japan). The average fluorescence intensity for each image was quantified using Fiji software (National Institutes of Health, USA).

### 2.11 Hydroxyphenyl fluorescein (HPF) staining

As previously described [[42], [43], [44]], the frozen tissue was sectioned (4–5 μm), immersed in 1 % acetone fixation solution at room temperature for 5 min, followed by PBS washing. 10 μM HPF (C3384, APExBIO, USA) was added to the samples and incubated in a dark humidified box at 4 °C–37 °C for 1 h. After PBS washing, the samples were stained with DAPI for 10 min. Images were acquired using a confocal microscope (SpinSR10, Olympus, Japan) with excitation/emission wavelengths of Ex/Em: 490/515 nm. The average fluorescence intensity of each image was quantified using Fiji software (National Institutes of Health, USA).

### 2.12 Singlet oxygen sensor green (SOSG) staining

As previously described [43,45], the frozen tissue was sectioned (4–5 μm), immersed in 1 % acetone fixation solution at room temperature for 5 min, followed by PBS washing. Following permeabilization, the sections were incubated with10 μM SOSG working solution (S36002, Thermo, USA) in a dark humidified box at 37 °C for 30 min. After washing, images were acquired using a confocal microscope (SpinSR10, Olympus, Japan) with excitation/emission wavelengths of Ex/Em: 504/525 nm. The average fluorescence intensity of each image was quantified using Fiji software (National Institutes of Health, USA).

### 2.13 Quantification of biochemical parameters(TG, TC, LDL-c, ALP, AST, and ALT)

The levels of alanine aminotransferase (ALT), aspartate transaminase (AST), and alkaline phosphatase (ALP) in serum were measured using a biochemical analyzer (Chemray 800, Rayto, China).

The frozen stored liver tissue levels of triglyceride (TG) (A110-1-1, Jiancheng, China), total cholesterol (TC) (A111-1-1, Jiancheng, China), and low-density lipoprotein cholesterol (LDL-c) (A113-1-1, Jiancheng, China) were measured using commercial kits according to the manufacturer's instructions.

### 2.14 8-Epi-PGF2α content measurement

The levels of 8-epi-PGF2α in serum and tissue were measured using a mouse 8-epi-PGF2α ELISA kit (E-EL-0041, Elabscience, China). For serum analysis, 50 μL of mouse serum and biotinylated antibody working solution were added to the pre-coated wells of a microplate and incubated at 37 °C for 45 min. After washing, HRP-conjugated working solution was added, and the plate was incubated at 37 °C for 30 min. After washing, TMB substrate solution was added, and the plate was incubated in the dark at 37 °C for 15 min. Finally, stop solution was added to each well, and the absorbance was immediately measured at 450 nm. For tissue analysis, tissue were homogenized in PBS using a tissue homogenizer, and the supernatant was collected after centrifugation. As described above, the 8-epi-PGF2α levels in the supernatant were measured by ELISA and further normalized to the total protein content of the tissue.

### 2.15 Ferritin, Interleukin-6 (IL-6), Interleukin-1β (IL-1β), and tumor necrosis factor α (TNF-α) content measurement

According to the manufacturer's instructions, ferritin levels in mouse liver (ELK10631, ELK Biotech, China) and liver inflammation markers (IL-1β (RE1074M, Reed Biotech, China), TNF-α (RE1060M, Reed Biotech, China), IL-6 (RE3186M, Reed Biotech, China)) were quantified using ELISA kits. Briefly, the liver tissues were homogenized, standard or sample was added to the wells pre-coated with detection antibody, followed by incubation at 37 °C for 60 min. The liquid was discarded, and after washing 6 times, 100 μL of HRP-labeled streptavidin was added and incubated for 30 min. Substrate solution TMB was added, and the plate was incubated in the dark at 37 °C for 15 min. Finally, stop solution was added, and absorbance was immediately measured at 450 nm using a microplate reader. The data were further normalized to the total protein content of the liver tissue.

### 2.16 Glutathione (GSH)/oxidized glutathione (GSSG) level measurement

GSH and GSSG levels were measured using a GSH and GSSG detection kit (S0053, Beyotime, China) and further normalized to the total protein content of the liver and spleen. As previously described [46], the working principle is that glutathione reductase reduces GSSG to GSH. The generated GSH reacts with the chromogenic substrate DTNB (5,5′-dithiobis-(2-nitrobenzoic acid)) to produce a yellow product, TNB (5-thio-2-nitrobenzoic acid), and GSSG. In this reaction system, the total glutathione concentration ([GSH] + 2 × [GSSG]) serves as the rate-limiting factor for color formation, and its concentration directly determines the amount of TNB produced. By measuring the absorbance at 412 nm (A412), the concentration of total glutathione can be quantified. To accurately measure GSSG, we first used a scavenger reagent to remove GSH from the sample, preventing interference with the measurement. In the presence of nicotinamide adenine dinucleotide phosphate (NADPH), GSSG is reduced by glutathione reductase to GSH, which is quantified via the above reaction mechanism. The GSH content in the sample can then be calculated by subtracting the GSSG amount from the total glutathione value. During sample processing, oxidation may lead to overestimation of GSSG. We harvested the tissues from *NRF2*^*−/−*^ and WT mice, and immediately froze them at −80 °C after measuring the magnetic susceptibility. Once all magnetic susceptibility measurements were completed, we thawed the tissues and immediately rinsed them with 0.9 % sodium chloride solution pre-cooled to 4 °C before they were homogenized in a cold solution containing 5 % orthophosphoric acid and 1 mM EDTA. The concentration of the sample was calculated based on a regression curve generated from gradient concentrations of GSSG and GSH standards. The detection limit of this method is 0.5 μM.

### 2.17 H_2_O_2_ level measurement

According to the manufacturer's instructions, H_2_O_2_ levels in the liver and spleen were measured using an H_2_O_2_ assay kit (A064-1-1, Jiancheng, China) and further normalized to the total protein content of the liver and spleen. Hydrogen peroxide (H_2_O_2_) reacts with molybdate to form a yellow molybdate-hydrogen peroxide complex. The reaction between H_2_O_2_ and molybdate produces a complex whose absorbance at 405 nm is linearly correlated with the concentration of H_2_O_2_. Using a standard curve, the concentration of H_2_O_2_ in the sample can be calculated based on the absorbance.

### 2.18 Malondialdehyde (MDA) content measurement

MDA levels in the liver and spleen were determined using an MDA assay kit (A003-1-2, Jiancheng, China). After tissue homogenization, tissue lysis buffer was added. The mixture was lysed at 4 °C, followed by centrifugation at 12000×*g* for 10 min to collect the supernatant. TBA dilution was prepared by dissolving 1.85 mg of TBA from the kit in 500 μL of buffer, and vortexed in the dark at 70 °C to promote dissolution. Then, the solution was mixed with 30 μL of antioxidant and diluted with water to a final volume of 1.5 mL to make the TBA working solution. Next, 250 μL of the tissue homogenate and 500 μL of the TBA working solution were mixed and boiled for 15 min. After cooling to room temperature, the mixture was centrifuged at 1000×g for 10 min, and the supernatant was transferred to a 96-well plate. Finally, 532 nm absorbance was measured using a microplate reader, and the data were further normalized to the total protein content of the liver and spleen.

### 2.19 Total iron and Fe^2+^ content measurement

Following the kit instructions, serum total iron levels were measured using a serum iron assay kit (A039-1-1, Jiancheng, China). For tissue analysis, total iron and Fe^2+^ levels in the liver and spleen were measured separately using the tissue total iron assay kit (A039-2-1, Jiancheng, China) and Fe^2+^ assay kit (AKIC004 M, BOXBIO, China). The tissue total iron and Fe^2+^ content were then normalized to the protein content.

### 2.20 Inductively coupled plasma (ICP) quantification of total iron content

Liver tissue was precisely weighed, dissolved, and centrifuged to collect the supernatant. Iron levels were quantified using a dual view inductively coupled plasma optical emission spectrometer (ICP6300, Thermo Fisher Scientific, USA). The data were expressed as micrograms of iron per gram of tissue wet weight.

### 2.21 Electron paramagnetic resonance (EPR) experiment

Free radicals were detected using an EPR spectrometer (Bruker EMX plus 10/12, USA) with instrument parameters set to 2 G field modulation, 100 G scanning range, and 5-mW microwave power. 5,5-dimethyl-1-pyrroline N-oxide (DMPO) (HY-107690, MCE, USA), 2,2,6,6-Tetramethyl-4-piperidone hydrochloride (TEMP) (T511, DOJINDO, Japan), and 5-tert-Butoxycarbonyl-5-methyl-1-pyrroline-N-oxide (BMPO) (B648752, aladdin, China) were used as spin traps for hydroxyl radicals, singlet oxygen, and superoxide anions, respectively. Additionally, the generation of free radical was validated by quenching the corresponding free radical with radical-specific scavengers, including salicylic acid (HY–B0167, MCE, USA), β-carotene (HY–N0411, MCE, USA), and superoxide dismutase (SOD) (HY-129064, MCE, USA). The corresponding EPR spectras were subsequently recorded and analyzed to confirm the formation of the target free radicals. Quantification of free radical content was achieved by analyzing the intensity of the EPR signals.

### 2.22 Quantification and statistical analysis

Power analysis was not performed to determine the sample size in mice. The sample size for each experimental study was based on previous research experience in mouse models. In this study, three researchers independently performed tissue section preparation, image acquisition, and other experimental procedures to ensure unbiased blinded investigations. During data processing, the researchers only had access to anonymized image data, which were used for quantitative analysis. Each experimental group included at least three mice. For each mouse, one tissue section was analyzed, and three independent representative regions were randomly captured from each section for analysis. All analyses were performed using GraphPad Prism 9.0 (GraphPad Software, USA), except for the EPR analysis, which was conducted using Origin 2021 (OriginLab, USA) and SQUID data fitting was performed using SquidLab software [35] in MATLAB R2022a (MathWorks, USA). For two independent groups in unpaired experiments, a two-tailed unpaired Student's *t*-test was used. For paired two-group experiments, a two-tailed paired Student's *t*-test was applied. ANOVA was used for analysis of experimental data from three or more groups, followed by Bonferroni-corrected multiple comparisons post-ANOVA. All data are presented as the mean ± standard error of the mean (SEM). *p* < 0.05 was considered statistically significant (∗*p* < 0.05, ∗∗*p* < 0.01, ∗∗∗*p* < 0.001), and ns indicates no statistically significant difference.

## 3 Results

### 3.1 *NRF2* deficiency leads to redox disturbances in liver and spleen

**Fig. 1:**
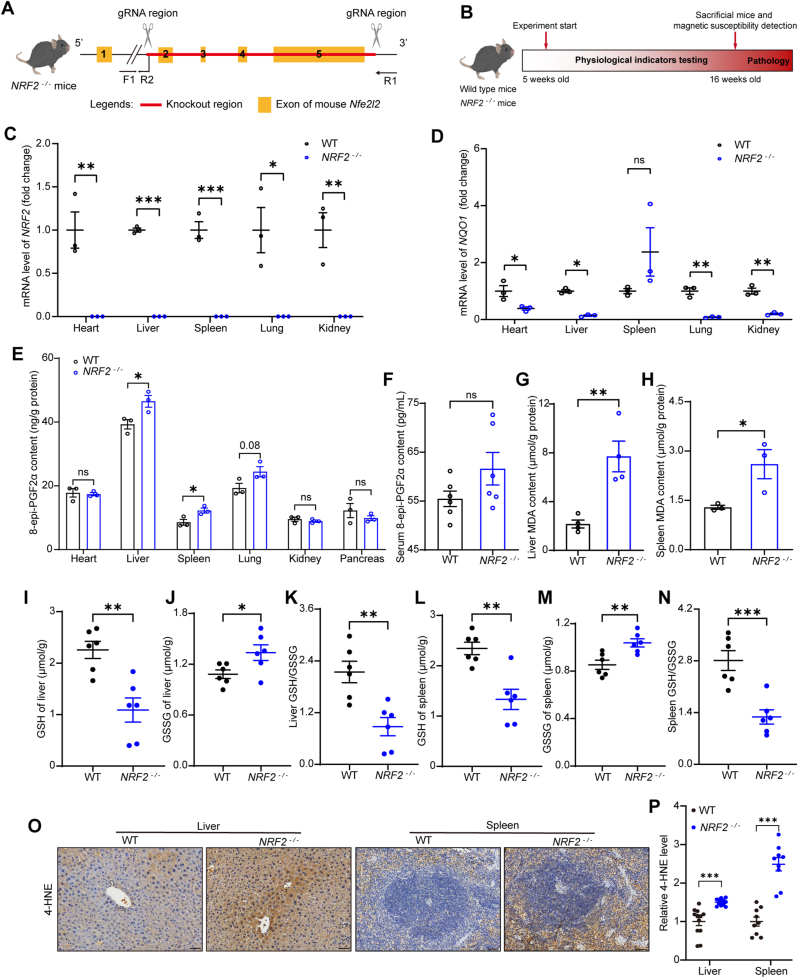
*NRF2* deficiency leads to altered redox homeostasis in the liver and spleen in mice. **(A)** Strategy for generating *NRF2*^*−/−*^ mice. **(B)** Experimental flow chart. **(C and D)** Validation of *NRF2* deficiency and downstream gene *NQO1* expression (n = 3 mice). **(E)** Levels of 8-epi-PGF2α, a clinical lipid peroxidation marker, in major organs of *NRF2*^*−/−*^ and wild-type mice (n = 3 mice). **(F)** Systemic circulating levels of 8-epi-PGF2α in *NRF2*^*−/−*^ and wild-type mice (n = 6 mice). **(G and H)** MDA content in the liver and spleen of *NRF2*^*−/−*^ and wild-type mice (n = 3–4 mice). **(I to K)** Levels of GSH and GSSG in the liver of *NRF2*^*−/−*^ and wild-type mice (n = 6 mice). **(L to N)** Levels of GSH and GSSG in the spleen of *NRF2*^*−/−*^ and wild-type mice (n = 6 mice). **(O and P)** Representative images of 4-HNE adduct staining and their quantification of the positive area in the liver and spleen of *NRF2*^*−/−*^ and wild-type mice (n = 3–4 mice, 3 microscopic fields for quantification, scale bar, 50 μm). Data are presented as mean ± SEM. ns = no statistically significant difference, ∗*p* < 0.05, ∗∗*p* < 0.01, ∗∗∗*p* < 0.001.

We used *NRF2* gene knockout mice (Fig. 1A) to examine the contribution of redox imbalance on the organ magnetic susceptibilities and functions (Fig. 1B). *NRF2* and its downstream target gene *NQO1* *(NAD(P)H quinone oxidoreductase 1)* were validated (Fig. 1C and D), which confirmed the loss of *NRF2*.

Next, we first assessed F2-isoprostanes (8-epi-PGF2α, also called F2-IsoP), a clinical marker of systemic oxidative stress, in the circulatory system and major organs, as it can effectively reflect changes in systemic oxidative stress [47]. The results showed that 8-epi-PGF2α levels were significantly elevated in the liver and spleen of *NRF2*^−/−^ mice, with a trend toward increased levels observed in lung tissue (p = 0.08) (Fig. 1E). However, no statistically significant changes were observed in systemic circulation (Fig. 1F). Additionally, measurements of MDA levels in the liver and spleen were consistent with these findings, further confirming the presence of lipid peroxidation induced by oxidative stress (Fig. 1G and H).

To assess changes in redox homeostasis, we measured the levels of reduced GSH and GSSG in the liver and spleen. The results demonstrated a significant shift in the GSH/GSSG ratio, indicating that *NRF2* deficiency induces abnormal redox states in these organs (Fig. 1I–N). We further assessed the level of 4-HNE adducts, an important marker of lipid peroxidation induced by oxidative stress [[36], [37], [38]], which were shown to be elevated in the liver and spleen of *NRF2*^*−/−*^ mice compared to wild-type mice, further indicating the accumulation of oxidative damage (Fig. 1O and P).

### 3.2 ROS may contribute to changes in liver and spleen magnetic susceptibility in *NRF2*^*−/−*^ mice

**Fig. 2:**
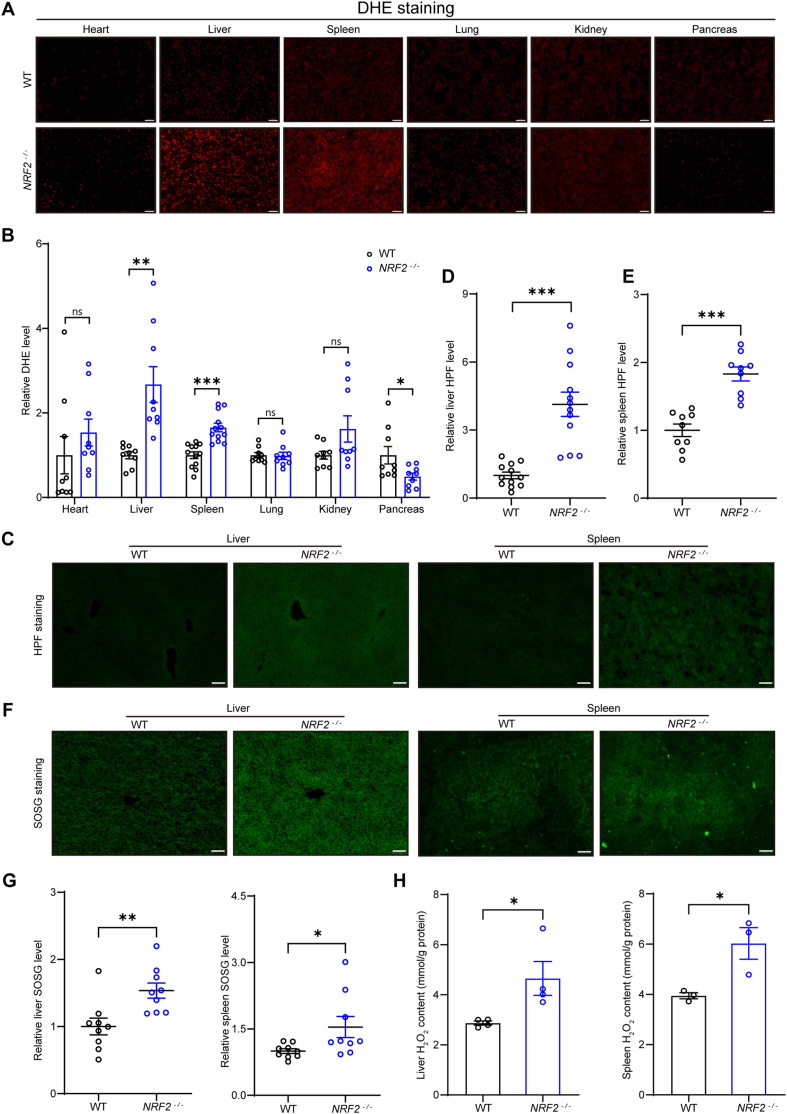
ROS levels in major organs of *NRF2*^*−/−*^ mice. **(A and B)** Representative images and quantification of superoxide anion staining in major organs of *NRF2*^*−/−*^ and wild-type mice using DHE, a fluorescent probe primarily targeting superoxide anions (n = 3 mice; 3 microscopic fields per mouse for quantification; scale bar, 50 μm). **(C to E)** Representative images and quantification of hydroxyl radical staining in the liver and spleen of *NRF2*^*−/−*^ and wild-type mice using HPF, a fluorescent probe specific for hydroxyl radicals (n = 4 mice; 3 microscopic fields for quantification; scale bar, 50 μm). **(F and G)** Representative images and quantification of singlet oxygen staining in the liver and spleen of *NRF2*^*−/−*^ and wild-type mice using SOSG, a fluorescent probe for singlet oxygen (n = 3 mice; 3 microscopic fields per mouse for quantification, scale bar, 50 μm). **(H)** Detection of H_2_O_2_ levels in the liver and spleen of *NRF2*^*−/−*^ and wild-type mice (n = 3–4 mice). Data are presented as mean ± SEM. ns = no statistically significant difference, ∗*p* < 0.05, ∗∗*p* < 0.01, ∗∗∗*p* < 0.001.

NRF2 plays a critical role in cellular antioxidant defense and is widely recognized as a key indicator of oxidative stress, while also functioning to maintain cellular redox homeostasis [29,48]. Using DHE staining, which primarily detects superoxide anions, we observed significantly elevated superoxide anion levels in the liver and spleen of *NRF2*^*−/−*^ mice (Fig. 2A and B). Furthermore, using the specific hydroxyl radical probe HPF [[42], [43], [44]], we found that hydroxyl radical levels were also significantly increased in the liver and spleen of *NRF2*^*−/−*^ mice (Fig. 2C–E). Additionally, analysis with the SOSG probe, a specific probe for singlet oxygen [43,45], revealed an increase in singlet oxygen levels in the liver and spleen of *NRF2*^*−/−*^ mice as well (Fig. 2F and G). Simultaneously, we measured the hydrogen peroxide (H_2_O_2_) levels in these organs and found that H_2_O_2_ levels in *NRF2*^*−/−*^ mice were significantly elevated compared to wild-type controls (Fig. 2H).

**Fig. 3:**
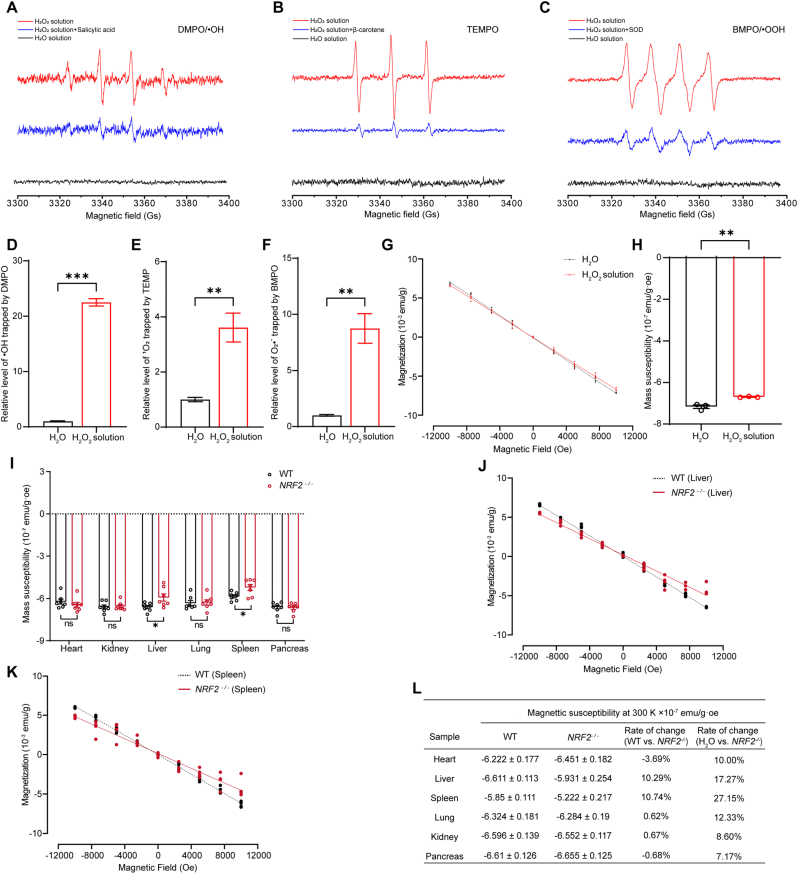
Free radicals may contribute to magnetic susceptibility changes in *NRF2*^*−/−*^ mice's liver and spleen. **(A)** EPR spectrum of the DMPO/•OH adduct formed after DMPO captures •OH. The generation of •OH was further confirmed by quenching the signal with salicylic acid. **(B)** EPR spectrum of TEMPO formed after TEMP captures ^1^O_2_. The generation of ^1^O_2_ was confirmed by quenching the signal with β-carotene. **(C)** EPR spectrum of the BMPO/•OOH adduct formed after BMPO captures O_2_•^-^. The generation of O_2_•^-^ was verified by quenching the signal with SOD. **(D to F)** EPR experiments were performed to detect and analyze the levels of free radicals trapped by spin traps in H_2_O_2_ solution and H_2_O (n = 4 times). **(G and H)** Representative M − H curves of ultrapure water and H_2_O_2_ and their magnetic susceptibility values. **(I)** Magnetic susceptibility of major organs in wild-type and *NRF2*^*−/−*^ mice measured at 300 K using SQUID (n = 7 mice). **(J and K)** Representative M − H (magnetization vs. magnetic field) curves for the liver and spleen of wild-type and *NRF2*^*−/−*^ mice measured at 300 K using SQUID. **(L)** Calculated magnetic susceptibility of major organs in wild-type and *NRF2*^*−/−*^ mice (n = 7 mice). Data are presented as mean ± SEM. ns = no statistically significant difference, ∗*p* < 0.05, ∗∗*p* < 0.01, ∗∗∗*p* < 0.001.

Although H_2_O_2_ itself is diamagnetic, it can easily decompose and generate some paramagnetic free radicals. To investigate the potential impact of excessive ROS on magnetic susceptibility, we used EPR to detect and quantify hydroxyl radicals (•OH), singlet oxygen (^1^O_2_), and superoxide anions (O_2_•^-^) in H_2_O_2_ solution. Although the overall free radical levels are low in H_2_O_2_ solution, they are detectable, which is in sharp contrast to those in ultrapure water (Fig. 3A–F). Moreover, we also performed EPR experiments with •OH scavenger (salicylic acid), ^1^O_2_ scavenger (β-carotene), and O_2_•^-^ scavenger (SOD) to verify their specificity. Our results show that after the addition of these scavengers, the target signals were significantly reduced, confirming that they originate from the specific reactive species (Fig. 3A–C). Further analysis of the magnetic susceptibility of H_2_O_2_ solutions containing trace amounts of hydroxyl radicals and superoxide anions revealed a significant increase compared to ultrapure water (Fig. 3G and H).

To avoid contamination such as iron, tissues such as the heart, liver, spleen, lung, kidney, and pancreas were collected using ceramic tools and washed with ultrapure water to remove any residual blood. No statistically significant differences were found between *NRF2*^*−/−*^ and wild-type mice in organ weight-to-body weight ratios (organ index) (Fig. S1). To further investigate the tissue magnetic differences of *NRF2*^*−/−*^ mice under near-physiological conditions, the M − H curves of heart, liver, spleen, lung, kidney, and pancreas tissue samples from *NRF2*^*−/−*^ and wild-type mice were measured immediately using an MPMS3 SQUID magnetometer at 300 K. The results showed that the magnetic susceptibilities of the heart, lung, kidney, and pancreas of *NRF2*^*−/−*^ mice did not differ significantly from those of wild-type mice (Fig. 3I).

However, the liver and spleen of *NRF2*^*−/−*^ mice exhibited significantly higher average mass magnetic susceptibility compared to wild-type mice, with an increase of approximately 10 % (*p* < 0.05), while the heart, lung, kidney, and pancreas did not (Fig. 3I–L). This observed difference in magnetic susceptibility in the liver and spleen is attributed to the accumulation of paramagnetic components. The magnetic susceptibilities of the organs we tested are different from those of ultrapure water (Fig. 3L). However, it is important to note that, despite these differences, the overall magnetic properties of all organs remained predominantly diamagnetic. It has been shown that a single paramagnetic molecule can cancel the diamagnetism of 100–1000 water molecules [49], but the concentrations of paramagnetic species are too low to overcome the predominant diamagnetism in tissues. Therefore, the diamagnetic property of water dominates the overall magnetic contribution, primarily due to the 60%–70% water content in the organs.

### 3.3 *NRF2* deficiency alters total iron, ferrous iron (Fe^2+^), and ferric iron (Fe^3+^) levels in the liver and spleen

**Fig. 4:**
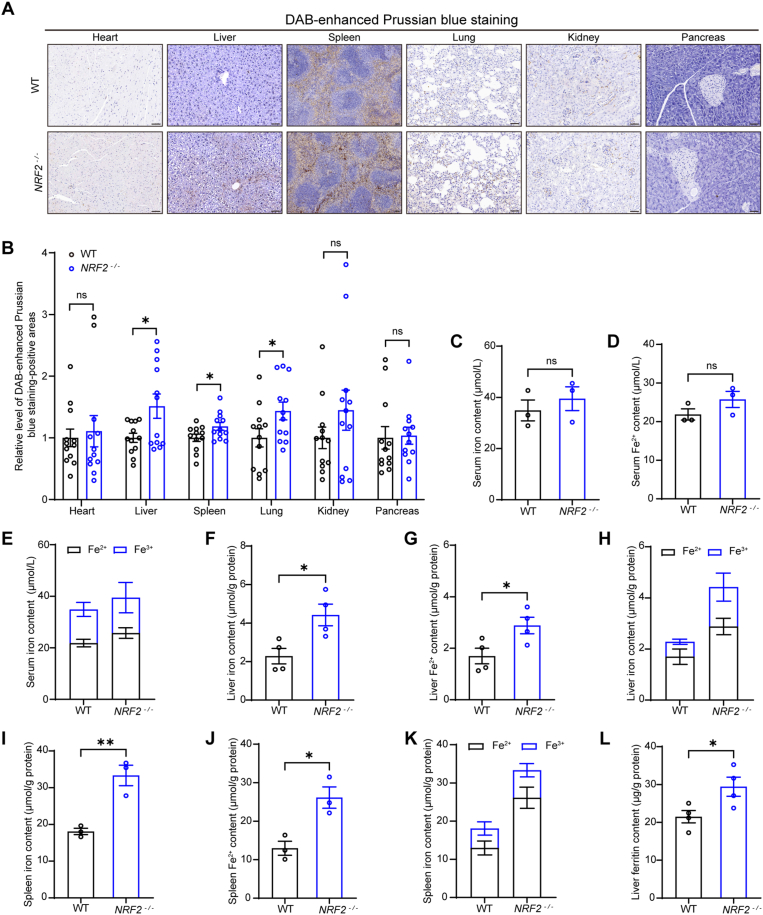
*NRF2*^*−/−*^ mice have increased iron content in their organs. **(A and B)** Representative images of DAB-enhanced Prussian blue staining in major organs of *NRF2*^*−/−*^ and wild-type mice and their quantification (n = 4 mice, 3 microscopic fields per mouse for quantification, scale bar, 50 μm). **(C to E)** Levels of iron, Fe^2+^, and Fe^3+^ in systemic circulation of *NRF2*^*−/−*^ and wild-type mice (n = 3 mice). **(F to H)** Levels of iron, Fe^2+^, and Fe^3+^ in the liver of *NRF2*^*−/−*^ and wild-type mice (n = 4 mice). **(I to K)** Levels of iron, Fe^2+^, and Fe^3+^ in the spleen of *NRF2*^*−/−*^ and wild-type mice (n = 3 mice). **(L)** Ferritin levels in the liver of *NRF2*^*−/−*^ and wild-type mice (n = 4 mice). Data are presented as mean ± SEM. ns = no statistically significant difference, ∗*p* < 0.05, ∗∗*p* < 0.01, ∗∗∗*p* < 0.001.

Numerous studies have shown that NRF2 plays a critical role in regulating intracellular iron homeostasis [[32], [33], [34]]. Therefore, in this study, we employed DAB-enhanced Prussian blue staining to examine the hearts, livers, spleens, lungs, kidneys, and pancreases of *NRF2*^*−/−*^ mice. The results revealed that, compared to wild-type mice, iron content was significantly increased in the livers, spleens, and lungs of *NRF2*^*−/−*^ mice (Fig. 4A and B). Additionally, analysis of total iron and Fe^2+^ levels in serum revealed no significant differences between *NRF2*^*−/−*^ mice and wild-type mice in systemic circulation (Fig. 4C–E). However, further analysis of total iron and Fe^2+^ levels in the spleens and livers of *NRF2*^*−/−*^ mice revealed significant increases in both, with altered relative proportions of Fe^3+^ and Fe^2+^ within these tissues (Fig. 4F–K). *NRF2* deficiency also led to an upregulation of iron storage proteins, causing iron accumulation in these tissues [34]. Consistently, our results showed a significant increase in liver ferritin content in *NRF2*^*−/−*^ mice (Fig. 4L).

### 3.4 Iron overload as a major contributing factor to magnetic susceptibility changes in the liver and spleen of *NRF2*^*−/−*^ mice

**Fig. 5:**
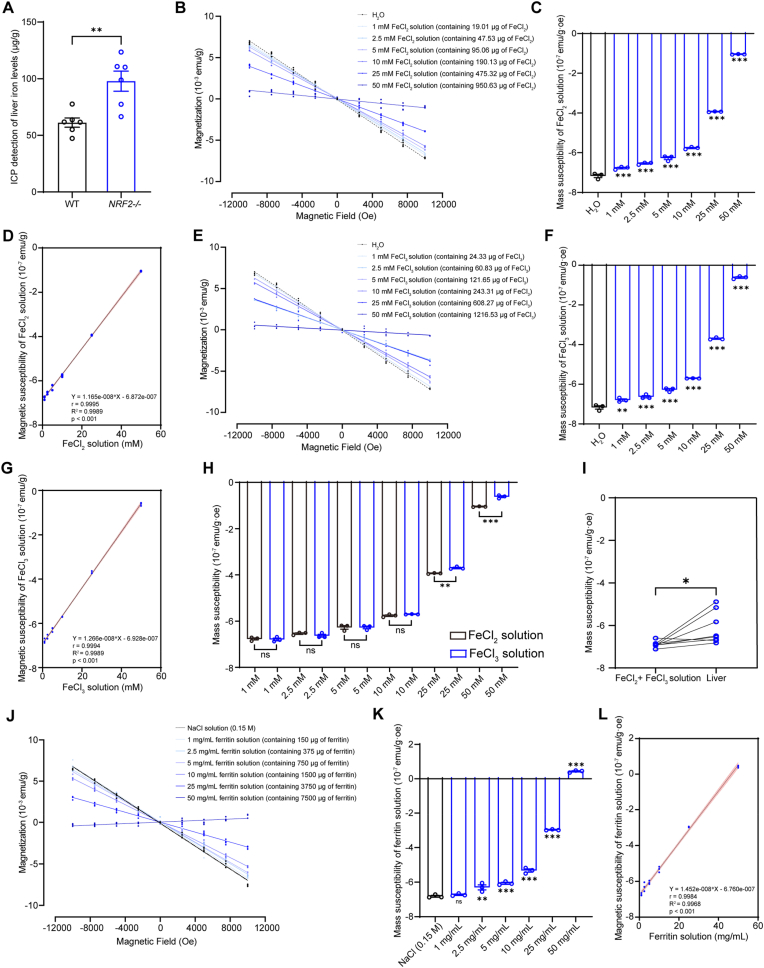
Magnetic susceptibilities of ferritin, Fe^2+^ and Fe^3+^ are directly correlated with their concentrations in solutions. **(A)** ICP quantification of total iron in the liver (n = 6 mice). **(B and C)** Representative M − H curves and magnetic susceptibility values (compared to H_2_O) of 150 μL H_2_O solution and 150 μL FeCl_2_ solutions at concentrations ranging from 1 to 50 mM (containing 19.01–950.63 μg of FeCl_2_). **(D)** Pearson correlation analysis of different FeCl_2_ solution concentrations and their corresponding magnetic susceptibility. **(E and F)** Representative M − H curves and magnetic susceptibility values (compared to H_2_O) of 150 μL H_2_O solution and 150 μL FeCl_3_ solutions at concentrations ranging from 1 to 50 mM (containing 24.33–1216.53 μg of FeCl_3_). **(G)** Pearson correlation analysis of different FeCl_3_ solution concentrations and their corresponding magnetic susceptibility. **(H)** Comparison of magnetic susceptibility values between FeCl_2_ and FeCl_3_ solutions. **(I)** Comparison of the magnetic susceptibility between FeCl_2_/FeCl_3_ mixed solutions with equivalent iron content in the liver. **(J and K)** Representative M − H curves and magnetic susceptibility values (compared to NaCl solution) of 150 μL NaCl solution and 150 μL ferritin solutions at concentrations ranging from 1 to 50 mg/mL (containing 150–7500 μg of ferritin). **(L)** Pearson correlation analysis of different ferritin solution concentrations and their corresponding magnetic susceptibility. Data are presented as mean ± SEM. ns = no statistically significant difference, ∗*p* < 0.05, ∗∗*p* < 0.01, ∗∗∗*p* < 0.001.

Previous studies have shown that iron plays a significant role in changes in organ magnetic susceptibility [50]. To precisely measure the total iron content in the liver, we employed the ICP method (Fig. 5A). We also measured the magnetic susceptibility of Fe^2+^ and Fe^3+^ solutions at various concentrations. The results demonstrated a significant increase in magnetic susceptibility with rising concentrations of Fe^2+^ and Fe^3+^, and Pearson correlation analysis revealed a strong positive correlation between their concentrations and magnetic susceptibility (Fig. 5B–G). When comparing magnetic susceptibilities at equivalent concentrations of Fe^2+^ and Fe^3+^, Fe^3+^ solutions exhibited significantly higher magnetic susceptibility than Fe^2+^ solutions at 25 mM and 50 mM, indicating magnetic susceptibility differences between these two oxidation states (Fig. 5H).

To better simulate the contributions of iron and its oxidation states to the magnetic susceptibility of *NRF2*^*−/−*^ mouse liver tissue, we prepared mixed Fe^2+^ and Fe^3+^ solutions based on their measured content in the liver (Fig. 4H). The results showed that the magnetic susceptibility of the mixed solution was (−689.4 ± 5.08)×10^−9^ emu/g·oe, which is higher than the ultrapure water ((−716.8 ± 8.591)×10^−9^ emu/g·oe), but lower than the liver magnetic susceptibility ((−61.36 ± 2.66)×10^−8^ emu/g·oe), indicating the presence of other paramagnetic components in the liver of *NRF2*^*−/−*^ mice (Fig. 5I), such as some paramagnetic free radicals.

Moreover, we also measured the magnetic susceptibility of ferritin solutions at various concentrations. Results showed that as ferritin concentration increased, the magnetic susceptibility of the solution rose significantly. At a concentration of 50 mg/mL, the ferritin solution exhibited overall paramagnetism ((43.05 ± 3.535)×10^−9^ emu/g·oe), while solutions in the range of 1–25 mg/mL range displayed overall diamagnetism (Fig. 5J and K). These results demonstrate that overall paramagnetic behavior only emerges when the components of paramagnetic components in a diamagnetic background exceeds a certain threshold. Pearson correlation analysis revealed a strong positive correlation between ferritin concentration and magnetic susceptibility levels (Fig. 5L).

### 3.5 *NRF2* deficiency causes impaired liver and spleen functions in mice

Excessive iron ions generate free radicals via the Fenton reaction, causing oxidative damage to cells and organs, which exacerbates the progression of various diseases [[51], [52], [53]]. Changes in Fe^2+^, Fe^3+^, and ROS levels in the liver and spleen of *NRF2*^*−/−*^ mice indicate significant alterations in the redox environment of these tissues. To assess the impact of redox imbalance and iron overload on mouse organs, we examined histological changes in major organs using H&E staining. The results showed slight damage to the liver and spleen of *NRF2*^*−/−*^ mice under conditions of redox imbalance and iron overload (Fig. S2).

**Fig. 6:**
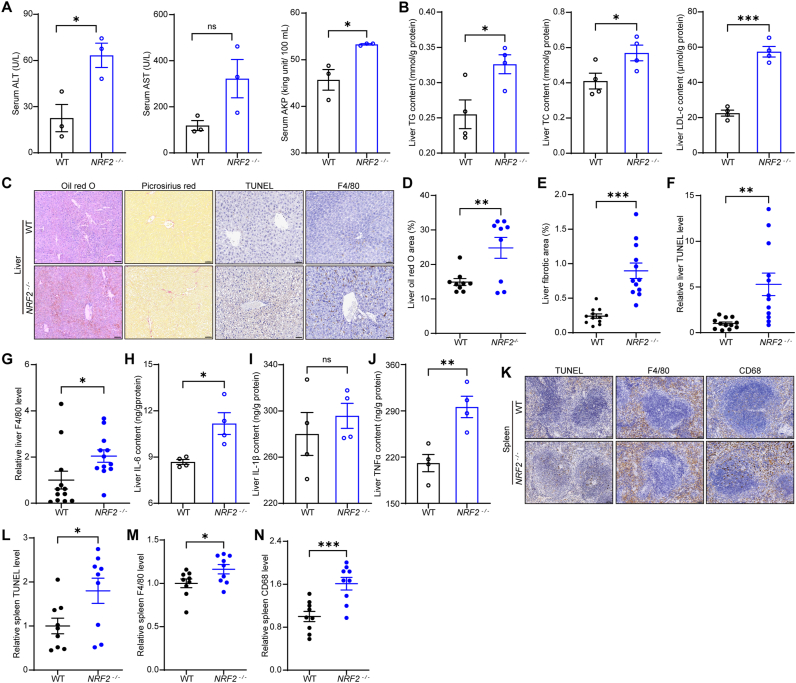
*NRF2*^*−/−*^ mice have increased liver and spleen damage. **(A)** Liver function levels of ALT, AST, and ALP in *NRF2*^*−/−*^ and wild-type mice (n = 3 mice). **(B)** Levels of TG, TC, and LDL-c in the liver of *NRF2*^*−/−*^ and wild-type mice (n = 4 mice). **(C to G)** Representative images and quantification of oil red O staining, Picrosirius Red staining, TUNEL staining, and F4/80 staining in the liver of *NRF2*^*−/−*^ and wild-type mice (n = 3–4 mice, 3 microscopic fields per mouse for quantification, scale bar, 50 μm). **(H to J)** Levels of IL-1β, IL-6, and TNFα in the liver of *NRF2*^*−/−*^ and wild-type mice (n = 4 mice). **(K to N)** Representative images and quantification of TUNEL staining, F4/80 staining, and CD68 staining in the spleen of *NRF2*^*−/−*^ and wild-type mice (n = 3 mice, 3 microscopic fields per mouse for quantification, scale bar, 50 μm). Data are presented as mean ± SEM. ns = no statistically significant difference, ∗*p* < 0.05, ∗∗*p* < 0.01, ∗∗∗*p* < 0.001.

We further evaluated liver function indices in *NRF2*^*−/−*^ mice and found that *NRF2* deficiency resulted in liver dysfunction (Fig. 6A). Measurements of TG, TC, and LDL-c in the liver revealed that *NRF2* deficiency resulted in abnormal liver lipid levels (Fig. 6B). Comprehensive analysis revealed that redox imbalance and iron overload in the liver of *NRF2*^*−/−*^ mice not only caused lipid accumulation but also led to fibrosis, apoptosis, and immune cell infiltration (Fig. 6C–G).

Previous studies have shown that diabetes can alter the iron content and magnetic susceptibility of the spleen [50]. Here we found no significant differences between 16-week-old *NRF2*^*−/−*^ mice and wild-type mice in body weight or blood glucose levels (Figs. S3A and B). No significant abnormalities were found in the HbA1c levels either, indicating that the average blood glucose of *NRF2*^*−/−*^ mice remained within the normal range in the recent past (Fig. S3C). Additionally, we assessed several metabolic-related parameters and found that the loss of *NRF2* had no obvious impact on glucose homeostasis or insulin tolerance in the mice (Figs. S3D–G).

**Fig. 7:**
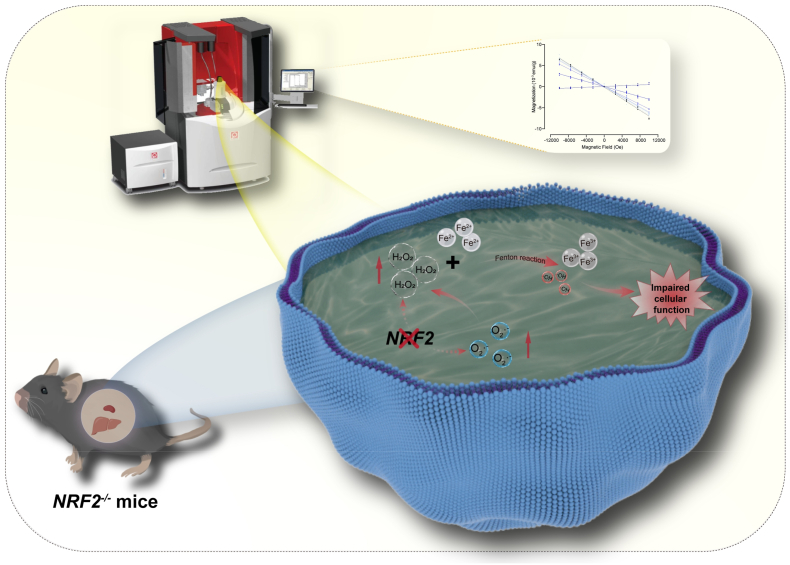
Redox imbalance-induced free radical and iron level elevations cause mice organ magnetic property and function alterations.

Furthermore, analysis of inflammatory factors showed a significant increase in inflammation levels in the liver of *NRF2*^*−/−*^ mice (Fig. 6H–J). Since the spleen plays an important role in immune regulation, we further analyzed inflammation-related markers in the spleen of *NRF2*^*−/−*^ mice. The results revealed that redox imbalance and iron overload promoted macrophage aggregation in the spleen and induced splenic cell apoptosis (Fig. 6K–N). Therefore, our study shows that redox imbalance induced free radical and iron level elevation not only can cause mice organ magnetic property changes, but also lead to function alterations in these tissues (Fig. 7).

## 4 Discussion

Redox reactions are essential for the existence of life and maintaining normal biological activities, whose imbalance is associated with aging and many diseases, including cardiovascular, diabetes and neurodegenerative diseases [5]. It has been shown that redox imbalance can be a major trigger of iron metabolism dysregulation [54]. Our study shows that redox imbalance in *NRF2*^*−/−*^ mice can cause increased free radical and iron levels, which cause magnetic property and function changes in their liver and spleen.

Our study confirms that the contents of paramagnetic Fe^2+^ and Fe^3+^ directly contribute to organ magnetic property changes in living organisms, but they are not the only reason. Our results show that redox dysregulation can enhance the magnetic susceptibility of *NRF2*^*−/−*^ mice by modulating both free radical production and iron deposition. It is well known that most free radicals are paramagnetic, but the contribution of free radicals to organ magnetic susceptibilities is often masked by iron in living organisms. In physiological conditions, the free radical levels are usually low. In contrast, iron is the most abundant essential trace element in our bodies. Our previous study showed that the iron-overload mice had a general increase in magnetic susceptibilities of all organs we examined, including the heart, liver, spleen, lung and kidney [50]. However, using *NRF2*^*−/−*^ mice, we found that the organ magnetic susceptibilities are not only determined by iron content, but also by ROS levels.

It should be mentioned that although *NRF2*^*−/−*^ caused redox and iron imbalances were expected, the differential alterations in the magnetic susceptibilities of the *NRF2*^*−/−*^ mice organs were unexpected, which highlights the fact that multiple factors are interconnected in living organisms. Our results indicate that ROS and iron generated a synergistic effect in affecting liver and spleen magnetic susceptibilities through the Fenton reaction. The Fenton reaction promotes the oxidation of Fe^2+^ to Fe^3+^ , generating an excess of free radicals, thereby exacerbating oxidative stress, while iron metabolism dysregulation further amplifies ROS production [51,52]. This ROS ―iron metabolism vicious cycle not only leads to increased iron deposition in organs but also exacerbates redox imbalance. This is likely the reason that contributes to the significantly increased ROS levels in the iron-rich organs, liver and spleen, of *NRF2*^*−/−*^ mice. Consequently, the magnetic susceptibilities and the normal functions of liver and spleen are both altered. In contrast, our previous study using iron-overload mice don't show much tissue specific effects because all their organs have increased iron content, which leads to a general increase in magnetic susceptibilities of all organs [50].

There are some limitations to our study, which should be further investigated in the future. First of all, to compare the magnetic susceptibilities of multiple tissues from both *NRF2*^*−/−*^ and WT mice using identical procedures side by side, we collected all tissues and performed magnetic susceptibility measurements before conducting other tissue analyses, including GSH/GSSG level measurement. Although this is not ideal because auto-oxidation of GSH to GSSG happens quickly, our results still showed obvious difference between *NRF2*^*−/−*^ and WT mice. Secondly, there are multiple mechanisms of Fe^2+^ oxidation to Fe^3+^, including Fenton reaction, pH-mediated processes and enzymatic oxidation reactions catalyzed by multicopper oxidases, which should be further explored in the future. For example, the oxidation of Fe^2+^ to Fe^3+^ is regulated by multiple factors, including pH [[55], [56], [57]] and enzymes [[58], [59], [60], [61]]. Studies have shown that at pH 3.5, the oxidation process is relatively slow, whereas at pH ∼7, the oxidation process is significantly accelerated, completing in just 8 min [55]. Even in the lysosomal environment with a low pH, Fe^2+^ can be rapidly oxidized to Fe^3+^, accompanied by the generation of free radicals [56,57]. Notably, compared to normal tissues, tumors typically exhibit lower pH, but this acidic environment may promote the release of more iron ions from ferritin, enhancing oxidative damage [57,62]. Therefore, the oxidation state of iron may change more rapidly in various cellular or subcellular environments and induce significant biological effects. Finally, *NRF2* deficiency may also trigger a cascade of downstream effects beyond free radicals and iron, involving changes in paramagnetic substances such as hemoglobin [63,64], transferrin [65], cytochrome *c* [66], and myoglobin [67]. Although the paramagnetism of these proteins primarily originates from iron, detailed changes in protein levels and their intertwined interactions with iron metabolism and redox processes in vivo, as well as their contributions to tissue magnetic susceptibilities and biological functions should also be further explored.

## 5 Conclusion

Our study found that the magnetic susceptibility of liver and spleen tissues in *NRF2*^*−/−*^ mice exhibited statistically significant differences compared to wild-type mice. Through a comprehensive analysis, we identified the pivotal roles of free radical levels and iron content in modulating magnetic susceptibility. While biological systems generally exhibit overall diamagnetism, the presence of paramagnetic or ferromagnetic substances within organs can induce variations in magnetic susceptibility. Notably, under conditions of redox imbalance, elevated free radical levels combined with the dysregulation of iron metabolism, not only change organ magnetic susceptibilities, but also induce organ dysfunction. This discovery not only provides novel theoretical insights into the relationship between redox regulation and the magnetic properties of living organisms, but also elucidates a key mechanism that contributes to tissue- and organ-specific sensitivity to magnetic fields. Furthermore, changes in magnetic susceptibility can also provide valuable information for clinical MRI applications, such as susceptibility-weighted imaging and quantitative susceptibility mapping.

## CRediT authorship contribution statement

**Chuanlin Feng:** Writing – review & editing, Writing – original draft, Validation, Methodology, Investigation, Formal analysis, Data curation. **Lei Zhang:** Writing – review & editing, Validation, Investigation. **Xiaoyuan Zhou:** Writing – review & editing, Validation, Investigation. **Shiyu Lu:** Writing – review & editing, Validation, Investigation. **Ruowen Guo:** Writing – review & editing, Validation, Investigation. **Chao Song:** Writing – review & editing, Writing – original draft, Validation, Resources, Investigation, Formal analysis. **Xin Zhang:** Writing – review & editing, Writing – original draft, Supervision, Project administration, Methodology, Funding acquisition.

## Declaration of competing interest

The authors have declared no conflict of interest.

## Data Availability

Data are available in the article or provided upon inquiry. https://doi.org/10.17632/h354f3j97v.5

## List of abbreviations

NRF2: nuclear factor erythroid 2-related factor 2
ROS: reactive oxygen species
SMFs: static magnetic fields
SWI: susceptibility-weighted imaging
QSM: quantitative susceptibility mapping
MRI: magnetic resonance imaging
SPF: specific pathogen-free
SQUID: superconducting quantum interference device
MPMS3: magnetic property measurement system 3
M − H curves: magnetization―magnetic field curves
χ_(mass)_: mass magnetic susceptibility
GTT: glucose tolerance test
ITT: insulin tolerance test
H&E: hematoxylin-eosin staining
4-HNE: 4-hydroxynonenal
DAB: 3,3′-diaminobenzidine
TUNEL: TdT-mediated dUTP Nick-End Labeling
TdT: terminal deoxynucleotidyl transferase
DHE: dihydroethidium
HPF: hydroxyphenyl fluorescein
SOSG: singlet oxygen sensor green
ALT: alanine aminotransferase
AST: aspartate transaminase
ALP: alkaline phosphatase
TG: triglyceride
TC: total cholesterol
LDL-c: low-density lipoprotein cholesterol
IL-6: interleukin-6
IL-1β: interleukin-1β
TNF-α: tumor necrosis factor α
GSH: glutathione
GSSG: oxidized glutathione
NADPH: nicotinamide adenine dinucleotide phosphate
MDA: malondialdehyde
EPR: electron paramagnetic resonance
DMPO: 5,5-dimethyl-1-pyrroline N-oxide
TEMP: 2,2,6,6-Tetramethyl-4-piperidone hydrochloride
BMPO: 5-tert-Butoxycarbonyl-5-methyl-1-pyrroline-N-oxide
SOD: superoxide dismutase
NQO1: NAD (P)H quinone oxidoreductase 1
8-epi-PGF2α: F2-isoprostanes
•OH: hydroxyl radicals
^1^O_2_: singlet oxygen
O_2_•: superoxide anions
H_2_O_2_: hydrogen peroxide
ICP: inductively coupled plasma
HbA1c: glycated hemoglobin
ANOVA: analysis of variance
SEM: standard error of the mean
