# Supplementary material for: Redox imbalance drives magnetic property and function changes in mice
Source: Redox Biol. 2025 Feb 21;81:103561. doi: 10.1016/j.redox.2025.103561 (PMC11910372; doi:10.1016/j.redox.2025.103561)
Supplement: Multimedia component 1 [file mmc1.docx]

**Supporting Information**

**Redox Imbalance Drives Magnetic Property and Function Changes in Mice**

**Chuanlin Feng^1,2^, Lei Zhang^1^, Xiaoyuan Zhou^3^, Shiyu Lu^1,2^, Ruowen Guo^1,2^, Chao Song^1^*, Xin Zhang^1,2,3^***

**
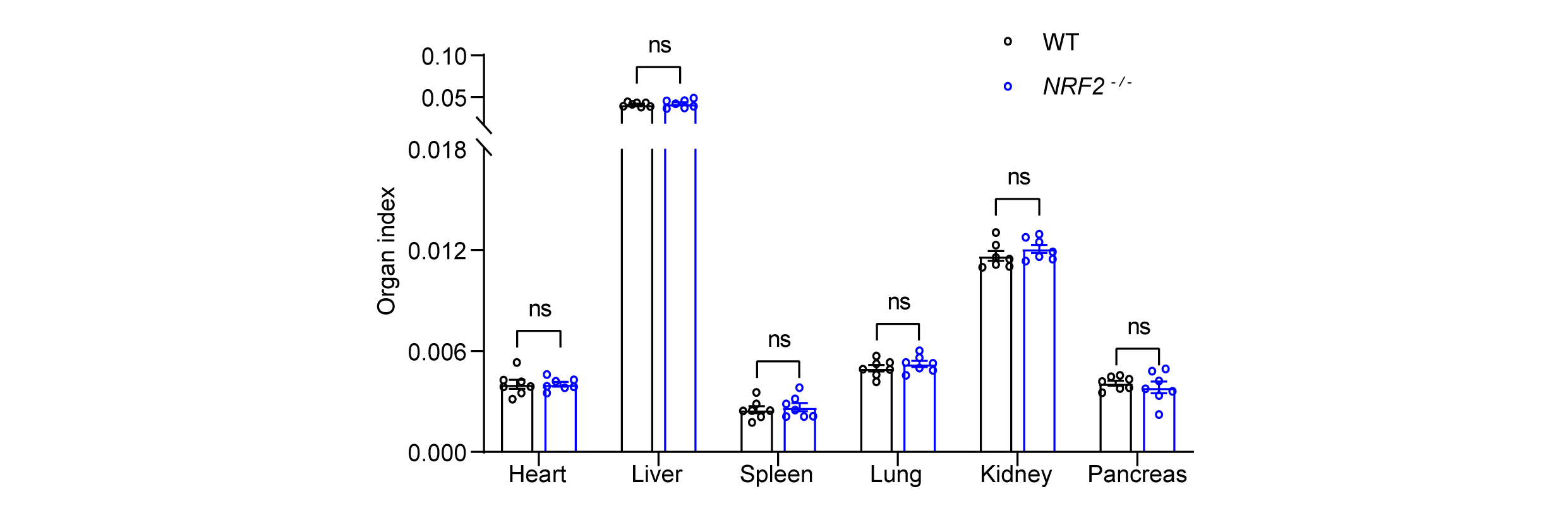
**

**Fig. S1 Organ indices of wild-type and *NRF2^-/-^* mice (n = 7 mice).** Data are presented as mean ± SEM. ns = no statistically significant difference.

**
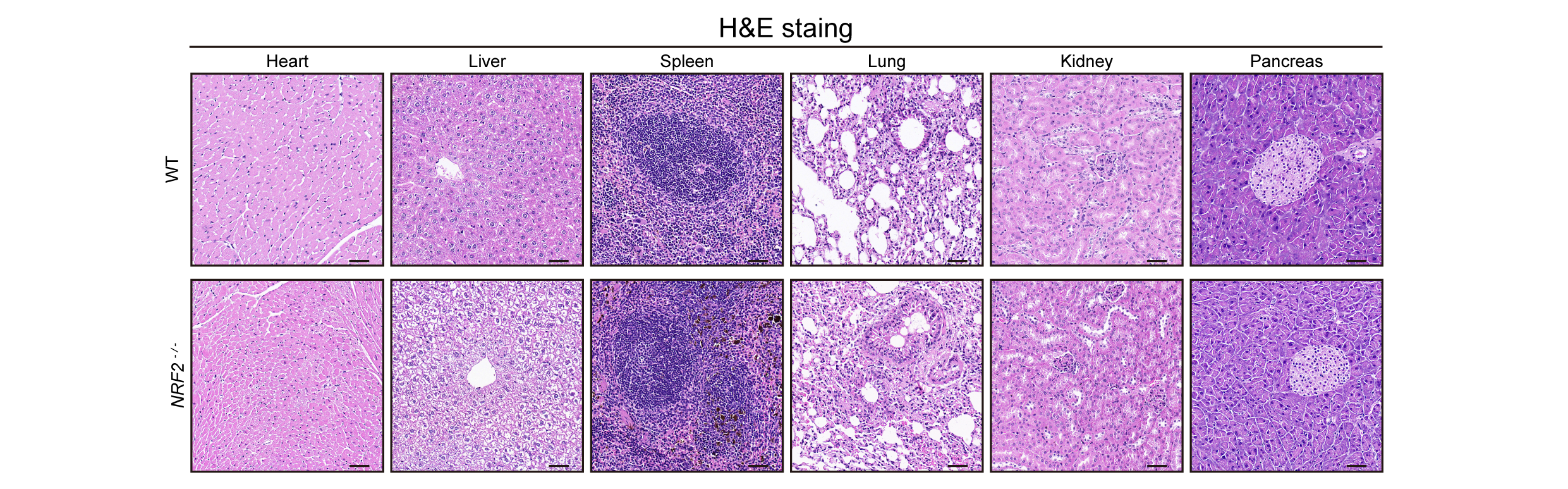
**

**Fig. S2 Representative H&E staining images of major organs from *NRF2^-/-^* and wild-type mice (scale bar, 50 μm).**

**
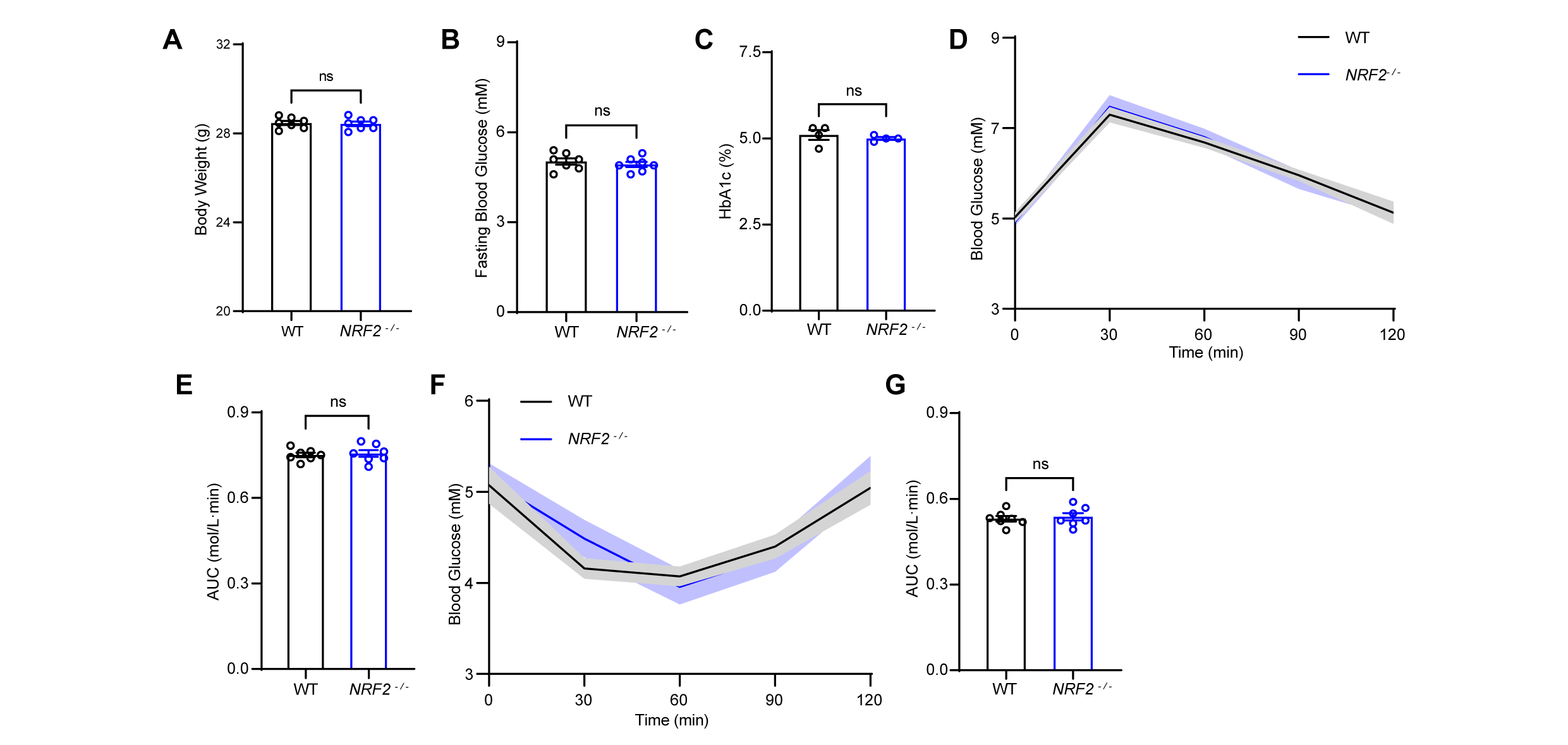
**

**Fig. S3 *NRF2* deficiency does not alter overall metabolic levels. (A)** Body weight (n = 7 mice). **(B and C)** Fasting blood glucose levels, and HbA1c levels in *NRF2^-/-^* mice (n = 7 mice). **(D and E)** Blood glucose trajectory and area under the curve during GTT (n = 7 mice). **(F and G)** Blood glucose trajectory and area under the curve during ITT (n = 7 mice). Data are presented as mean ± SEM. ns = no statistically significant difference.
